# *Streptococcus canis* Bacteremia in a Renal Transplant Recipient

**DOI:** 10.1177/2324709619834592

**Published:** 2019-04-01

**Authors:** Syed M. H. Zaidi, Ambika Eranki

**Affiliations:** 1 State University of New York Upstate Medical University, Syracuse, NY, USA

**Keywords:** *Streptococcus canis*, renal transplant, bacteremia

## Abstract

A middle-aged man presented with fever and shortness of breath. He had significant history of congestive heart disease and received deceased donor renal transplant 2 years prior to presentation. He was febrile and found to have sepsis. His initial blood cultures grew *Streptococcus canis. Streptococcus canis* causes rare infection in humans, and this is most likely the first case in the renal transplant population.

## Brief History of the Present Illness

A 62-year-old man presented with complaints of fever, chills, nausea, and vomiting from 3 days and progressive dyspnea from the past 2 days. In the emergency room, he was found to be febrile at 39.4°C, hypotensive blood pressure 90/60 mm Hg, and hypoxic (oxygen saturation 88%). He was started on piperacillin/tazobactam as a broad-spectrum empiric antibiotic.

The patient received a deceased donor renal transplant 2 year prior to presentation. His posttransplant course was complicated with CMV (cytomegalovirus) viremia, and he was on the following posttransplant regimen: belatacept 675 mg q 28 days, prednisone 5 mg daily, and prophylactic valganciclovir. His past medical history is also significant for congestive heart failure, atrial fibrillation, pituitary adenoma, hypertension, hypothyroidism, diabetes mellitus, and obstructive sleep apnea.

He used to get lower limb superficial skin ulcers and relates it to multiple factors including diabetes mellitus, chronic pedal edema, and use of prednisolone. He lives at home with his wife. His wife runs a dog obedience school from home, and they currently have 6 dogs. The dogs are very close to him and they often lick his skin.

On our examination, blood pressure was 130/90 mm Hg, pulse rate was 90 beats per minute, temperature was 38.3°C, and respiratory rate was 22 breaths per minute. The patient was noted to have pedal edema and multiple ecchymoses at venipuncture sites. Chest examination was positive for bilateral fine crepitations at bases, and abdomen was mildly distended. On cardiac examination, he was found to have irregularly irregular rhythm; no murmurs were appreciable. Neurologic examination was grossly intact. Skin examination showed a healed ulcer on the medial aspect of left leg.

On admission, patient’s white blood cell count was 3.5 × 10^9^/L, hemoglobin 10.2 mg/dL, platelet 81 × 10^9^/L, creatinine 2.5 mg/dL, blood urea nitrogen 35 mg/dL, and lactate 3.2 mmol. The chest X-ray showed bilateral congestion suggestive of pulmonary edema. His blood cultures from admission grew *Streptococcus canis* in aerobic and anaerobic broths; isolate was penicillin susceptible with minimum inhibitory concentration of 0.023.

His hypotension and fever improved after initial treatment. Shortness of breath gradually improved over 3 days with aggressive diuresis. On day 2 when the blood cultures result came his antibiotics were switched to ceftriaxone. Transthoracic echocardiogram did not show any vegetations, and transesophageal echocardiogram was not done because of the transient nature of bacteremia. The patient responded well to appropriate antibiotic therapy, and repeat blood cultures after 48 hours were negative. His creatinine levels improved and came back to his baseline of 1.3 mg/dL on day 3.

He was discharged home with intravenous ceftriaxone 2 g once daily for a total duration of 14 days. He was doing well on follow-up visit after 2 weeks, and the treatment was discontinued.

## Discussion

Bloodstream infections are a major reason of mortality and morbidity after transplantation.^1^ In solid organ transplant recipients, blood stream inflection–related mortality can be up to 50% when bacteremia is associated with septic shock.^2^

Several case reports have shown the transmission of zoonoses to humans during and after solid-organ and hematopoietic stem cell transplantations.^3^ Most zoonoses occur as a primary infection after transplantation, and immunocompromised patients are more likely to experience significant morbidity and mortality from these infections.^4^

*Streptococcus canis* is part of the pyogenic Lancefield group G family and has a long known history in veterinary medicine. It can cause respiratory, cutaneous, genital, and urinary infections in several animal species and reportedly causes bacteremia and mastitis.^5,6^

The group G β-hemolytic streptococci consist of *Streptococcus dysgalactiae* subspecies *equisimilis, S milleri, S canis*, and *S intestinalis*. Humans are the reservoirs of *S dysgalactiae* subspecies *equisimilis* and *S milleri*, whereas dogs and pigs are the reservoirs of *S canis* and *S intestinalis*, respectively.

In humans, *S canis* has been reported to cause mild noninvasive infections to severe sepsis.^7^ *Streptococcus canis* infections are rare and constitute only 1% of all streptococcal infections. Most patients have animal contact, especially dogs.^8,9^ It is difficult to distinguish β-hemolytic Lancefield group G *S dysgalactiae* and *S canis* when only Lancefield typing is performed. To accurately identify *S canis*, phenotypic testing and 16S rRNA gene sequencing is required. Therefore, there may be an underestimation of the true number of infections.^10^

Most of the reported cases were associated with history of dog contact and found to have skin wounds. In those cases, blood or wound cultures were positive with *S canis*.^10^ In our case, the patient had chronic pedal edema and history of recurrent superficial skin ulcers on the legs and close contact with dogs. At the time of presentation, he did not have open wound or infected ulcer, which makes the case unique.

Bert and Lambert-Zechovsky described a similar case of *S canis* bacteremia in a patient with exposure to a dog, and the apparent focus of infection was healed ulcers due to varicose veins on the legs. However, that patient was immunocompetent unlike our patient who was severely immunocompromised.^11^

Based on our review of the available literature, there have been no reported cases of *S canis* bacteremia in transplant recipients.

This case elucidates a rarely reported cause of bacteremia in a profoundly immunocompromised patient population and highlights the importance of obtaining a thorough history from every patient, as this can provide important epidemiologic clues about the underlying diagnosis.
